# Walking

**Published:** 1872-04

**Authors:** 


### WALKING

Briskly, with an exciting object or pleasurable interest ahead, is the most healthful of all forms of exercise except that of encouragingly remunerative, steady labor in the open air; and yet multitudes in the city, whose health urgently requires exercise, seldom walk when they can ride, if the distance is a mile or more. It is worse in the country, especially with the well-to-do; a horse or carriage must be brought to the door even if less distances have to be passed. Under the conditions first named walking is a bliss; it gives animation to the mind, it vivifies the circulation, it paints the cheek and sparkles the eye, and wakes up the whole being, physical, mental, and moral. We know a family of children in this city who, from the age of seven, had to walk nearly two miles to school, winter and summer; whether sleet, or storm, or rain, or burning sun, they made it an ambition never to stay away from school on account of the weather, and never to be "late;" and one of them was heard to boast that in seven years it had never been necessary to give an "excuse" for being one minute behind the time, even although in winter it was necessary to dress by gaslight. They did not average two days' sickness in a year, and later they thought nothing of walking twelve miles at a time in the Swiss mountains. Sometimes they would be caught in drenching rains, and wet to the skin; on such occasions they made it a point to do one thing, — let it rain, — and trudged on more vigorously until every thread was dry before they reached home.

There is no unmedicinal remedy known to men, of more value in the prevention of

CONSTIPATION, than a few miles' joyous walking; let one follow it up a week — a walk of two or three miles in the forenoon, and as much in the afternoon — and except in rare cases, when a longer continuance may be made, the result will be triumphant; and yet nine persons out of ten would rather give a dollar a bottle for some nauseous drops or poisonous pills, than take the trouble to put in practice the natural remedy of walking. Nor is there an anodyne among all the drugs in the world, which is the hundredth part so efficacious in securing refreshing, healthful, delicious,

GLORIOUS SLEEP, as a judicious walk. To be judicious it should not be continued so long as to make the person feel fagged out; this can be prevented by turning back when there is only a little tiredness; then after resting awhile on a sofa or bed, without closing the eyes, repeat the process, until in the course of the day several hours have been spent in the open air. Then after a light, early supper of a piece of bread and butter and a cup of tea, spend several hours in pleasant conversation or in games and amusements, not retiring before ten o'clock, going to bed with warm feet, not sleeping any after five in the morning, yet not leaving the bed until you feel like it, and not sleeping a single moment in the day-time; and if in less than one week you cannot sleep deliciously for several hours unbrokenly, there is something the matter with the brain, and you are a candidate for the asylum, unless you take prompt and able medical advice.

No drug, or drop, or pill, or potion, can by any possibility give refreshing, invigorating, healthful sleep; they all, without exception, aggravate the

SLEEPLESSNESS; and it is greatly to be deplored that such multitudes, especially in cities, are constantly resorting to the drug stores for something to make them sleep, — even young men and women.

Nervous people are specially inclined to take something to make them sleep, with the effect to keep them nervous, and prevent refreshing sleep year after year, thus embittering their own and the lives of those in the same household. In all such cases there is an excess of nervous energy, and that too of an unhealthful character; there is too much steam aboard; the remedy is to work it off in pedestrian excursions.

In walking, as in all other vigorous forms of exercise, the object fails to be accomplished, sickness is induced, and not unfrequently life itself is lost, for the want of taking a common-sense precaution to cool off slowly after the exercise, before a good fire in winter, in a warm room of seventy-five degrees in summer, with every window and door shut, no garment removed, even hat or glove, for five minutes; then shawl or overcoat by degrees, so as to cool off very slowly in the course of half an hour. It is no exaggeration to say that thousands of lives are sacrificed every year from failing to

COOL OFF SLOWLY

after the exercise has been taken. It is not so much going out of doors and being out of doors that gives people coughs, colds, and consumption, as the getting of these things after coming into the house, by cooling off too quickly.

